# Staging CT chest for cT1a renal masses: Does it change management?

**DOI:** 10.1002/bco2.70068

**Published:** 2025-09-22

**Authors:** Sanjana Ilangovan, Hannah Warren, Federica Sordelli, Thet Paing Oo, Pyae Phyo Tun, Prasad Patki, Faiz Mumtaz, Ravi Barod, Axel Bex, Maxine Tran

**Affiliations:** ^1^ Specialist Centre for Kidney Cancer Royal Free Hospital NHS Trust London UK; ^2^ Queen Elizabeth University Hospital Glasgow UK; ^3^ Division of Surgery and Interventional Sciences University College London London UK

**Keywords:** chest computed tomography, kidney cancer, pulmonary metastases, renal cell carcinoma, T1a renal tumours

## Abstract

**Objectives:**

Baseline staging investigations for renal masses invariably include a CT of the chest. However, EAU guidelines have given a weak recommendation that CT chest can be omitted in incidental T1a tumours (≤4 cm) without systemic symptoms, due to the low incidence of pulmonary metastases. This study aimed to assess if a baseline staging CT chest has been clinically useful in a cohort with T1a renal tumours.

**Methods:**

Consecutive patients with solid and cystic cT1a renal tumours were prospectively screened for eligibility to the NEST study (ISRCTN 18156881) at a single tertiary referral centre multidisciplinary team meeting (MDT). Four hundred consecutive eligible patients between 28/05/2019 and 13/01/2021 were included in this study. Electronic records were reviewed retrospectively for follow‐up data. Seventeen patients with incomplete follow‐up data were excluded.

**Results:**

Of 383 included patients (63% male, median age 65 years, median tumour diameter 2.4 cm), 264 (69%) had a baseline CT chest as part of their clinical staging investigations. No thoracic renal metastases were diagnosed. Abnormalities were reported in 37/264 cases (14%), including indeterminate lung lesions in 32 patients that were deemed benign on further investigations, three synchronous primary lung tumours, one pre‐existing mesothelioma and one pleural effusion related to known renal failure.

**Conclusion:**

CT chest is of limited value in clinical staging investigations for cT1a renal tumours and has a negligible impact on subsequent renal tumour management. Rather, it triggered further investigations and follow‐up for 14% of incidentalomas and ultimately detected concurrent incidental primary lung tumours in 1% of patients.

## 1 INTRODUCTION

Renal cell carcinoma (RCC) is the 7th most common malignancy worldwide, accounting for approximately 2–3% of all cancer‐related deaths.^1^, ^2^ RCC has a high risk of metastatic spread,^3^ with the lungs being the most common site of metastasis.^4^ As a result, staging at diagnosis traditionally involved a CT scan of the chest, abdomen and pelvis to assess both the primary tumour and potential metastatic sites.

With the widespread use of cross‐sectional abdominal imaging in modern medical practice, the incidental detection of cT1a tumours has increased significantly.^5^ However, in the case of small renal masses (cT1a), defined as tumours measuring 4 cm or less, the risk of pulmonary metastasis is low, estimated at less than 1%, especially in the absence of systemic symptoms or other risk factors.^6^, ^7^ Despite the low metastatic risk in this group, incidental tumours have historically prompted further evaluation with CT thorax to exclude the presence of metastatic disease. This sometimes necessitates an additional visit for a separate CT thorax to complete staging, contributing to patient inconvenience, resource utilisation, increased radiation exposure, delayed definitive treatment and increased healthcare costs.^8^ Moreover, while the detection rate of lung metastases in this population remains low, some studies have reported a high frequency of incidental findings on CT thorax, raising questions about the true clinical utility of routine thoracic imaging in this setting.^9^

Despite these concerns, routine staging with CT thorax remains a standard practice in many institutions, including our centre, where all new suspected cancer cases must be discussed at a specialist tumour board or multidisciplinary team (MDT) meeting. The results of initial imaging play a crucial role in guiding management decisions.

In 2023, however, the European Association of Urology (EAU) guidelines introduced a significant update regarding the staging of cT1a renal masses. Specifically, the guidelines issued a weak recommendation to omit chest CT in patients with incidental cT1a renal tumours, acknowledging the low probability of metastatic events in this population.^5^ This shift reflects growing evidence that routine thoracic imaging in these cases may offer minimal clinical benefit while exposing patients to unnecessary investigations for benign incidental findings, radiation and healthcare costs.

In light of these evolving guidelines, this study aimed to evaluate the use of CT thorax in the staging of cT1a renal masses at our high‐volume tertiary referral centre prior to the guideline change. Specifically, we sought to assess whether the results of CT thorax influenced clinical decision‐making.

## 2 METHODS

Consecutive patients with a solid or cystic small renal mass referred to the Royal Free Hospital Specialist Centre for kidney cancer multidisciplinary team meeting were screened for eligibility to a longitudinal cohort study, Nephron Sparing Treatment for small renal masses (NEST) (UK HRA REC 19/EM/0004). The NEST study is an ongoing randomised controlled trial comparing active treatment options, which are percutaneous cryoablation and robot‐assisted partial nephrectomy for T1a renal tumours. In brief, all patients with a newly diagnosed clinical T stage 1a renal tumour were eligible for inclusion.

Four hundred eligible patients for this study were identified from this cohort between May 2019 and January 2021. The electronic medical records of the patients were reviewed retrospectively. A total of 17 patients were excluded from the study due to incomplete follow‐up data. Collected data included patient demographics, prevalence of pre‐existing thoracic pathology, tumour characteristics, imaging data and oncological outcomes. Study results are described qualitatively and quantitatively as a proportion of affected patients. The mean time from diagnosis to treatment of renal tumour was 152 days.

## 3 RESULTS

A total of 383 patients with solid and cystic cT1a renal masses were included in this study. The demographic and pathological data are detailed in Table 1.

**TABLE 1:** Demographics and pathological data of 383 patients with cT1a included in this study.

	Number of patients	Percentage (95% CI)	Median	Interquartile range (IQR)
**Gender**				
*Male*	242	63.19 (58.14–68.03)		
*Female*	141	36.81 (31.97–41.86)		
**Age**			65	56–73
**Pre‐existing thoracic pathology**				
*COPD*	13	3.4 (1.8–5.7)		
*Asthma*	15	3.9 (2.2–6.4)		
*COVID*	1	0.26 (0.01–1.45)		
*Bronchiectasis*	3	0.78 (0.16–2.27)		
*Lung malignancy*	2	0.52 (0.06–1.87)		
*Mesothelioma*	1	0.26 (0.01–1.45)		
*Pulmonary embolism*	3	0.78 (0.16–2.27)		
**Tumour diameter (mm)**			24	17–30

Two hundred and sixty‐four out of 383 patients (69%) had a baseline staging CT chest available at the time of discussion at the specialist tumour board/MDT meeting. The remaining 119 patients (31%) had incidental findings of small renal masses noted on the CT abdomen and pelvis without CT thorax. As the NEST study was a pragmatic study where patients were managed according to standard care, typically, patients having active treatment would have completion staging with CT chest at baseline, while patients embarking on surveillance would have CT chest combined with their first interval surveillance scan at 6 months. The characteristics of the patient group that had a baseline CT thorax and the group that did not have a baseline CT chest were compared in Table 2.

**TABLE 2:** Comparison of characteristics of the patient cohort with a baseline CT thorax and those without a baseline CT thorax.

	Patients with CT thorax (Total = 264)				Patients without CT thorax (Total = 119)			
	Number of patients	Percentage (95% CI)	Median	IQR	Number of patients	Percentage (95% CI)	Median	IQR
**Gender**								
*Male*	164	62.12 (56.66–67.58)			79	66.38 (62.22–70.54)		
*Female*	100	37.88 (32.42–43.34)			40	33.62 (29.46–37.78)		
**Age**			66	56–73			64	56–73
**Pre ‐ existing lung conditions**								
*COPD*	10	3.78 (3.57–3.99)			3	2.52 (2.30–2.73)		
*Asthma*	12	4.55 (4.34–4.76)			3	2.52 (2.30–2.73)		
*COVID*	1	0.38 (0.17–0.59)			0	0		
*Bronchiectasis*	2	0.76 (0.55–0.97)			1	0.84 (0.77–0.91)		
*Lung malignancy*	2	0.76 (0.55–0.97)			0	0		
*Mesothelioma*	1	0.38 (0.17–0.59)			0	0		
*Pulmonary embolism*	3	1.13 (0.92–1.34)			0	0		
**Tumour size (diameter)**			26	19–31			20	15–29

Of the 264 patients who had a baseline CT chest performed, 156 patients (59%) required a separate, additional visit to the radiology department for that imaging episode. Across the 264 CT chest studies, 37 (14%) demonstrated abnormalities, leading to further investigations in 19 (51%) patients who underwent additional interval imaging, typically a repeat CT chest after three months to assess for progression or resolution, and 8 (22%) were referred to a lung MDT for imaging review. The remaining 10 (37%) patients did not require further investigations.

Of the eight cases who were referred to lung MDT, six subsequently underwent further invasive investigations, including endobronchial ultrasound (EBUS) bronchoscopy and/or needle biopsy, which confirmed benign nodules in three patients and identified synchronous primary lung tumours in the other three patients. The two patients who did not have invasive investigations had their scans reviewed and deemed not to require follow‐up.

**FIGURE 1:**
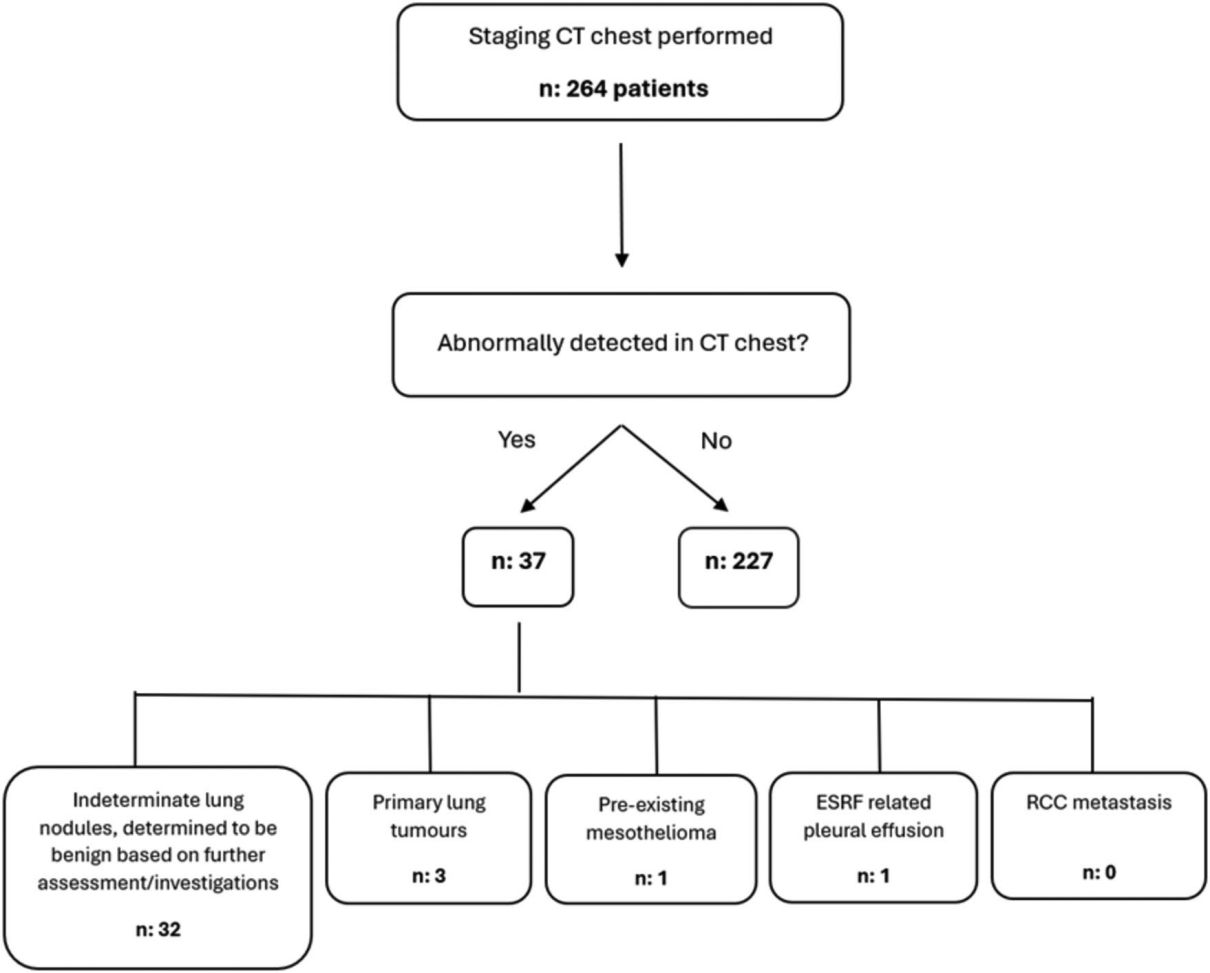
Flow diagram of outcomes of staging CT chest performed in 264 patients.

Overall, no metastases secondary to RCC were detected. Three patients (1.1%) were found to have synchronous primary lung tumours. Additionally, 32 patients were diagnosed with benign lung nodules, 1 with pleural effusions related to known end‐stage renal failure and 1 with pre‐existing mesothelioma (Figure 1).

The detection of lung malignancy led to a change in renal tumour management in all three patients with concurrent primary lung tumours. They were put on the active surveillance pathway whilst they underwent surgical resection of their lung tumours based on imaging evidence. Two of these patients eventually underwent cryoablation, whilst the remaining patient had surgery for their renal tumour.

## 4 DISCUSSION

The EAU guidelines have suggested that baseline staging CT chest can be omitted in patients with cT1a renal masses due to the low risk of pulmonary metastasis. This recommendation is based on large studies demonstrating that the risk of lung metastasis in patients with incidental small renal masses is <1%.^6^, ^7^, ^10^ However, our local multidisciplinary team (MDT) referral criteria currently recommend performing a staging CT chest before the initial MDT discussion. In this study, we evaluated the clinical use and outcomes from routine CT chest staging for cT1a renal masses in a high‐volume tertiary referral centre.

Our findings showed that no thoracic metastases secondary to renal tumours were identified on the staging CT chests. Our study findings corroborate with the study by Voss et. al that influenced the recommendation to omit CT chests by EAU guidelines, which found none of the three positive chests CT scans (out of 391) in their cT1a cohort had lung metastasis.^10^ Staging CT chests required 156 patients (59%) to make an additional visit to the radiology department for this as a separate imaging encounter, potentially increasing patient anxiety and burdening healthcare resources.^8^ Moreover, CT chest scans carry a significant radiation exposure, with an average effective dose of 7 mSv, 70 times higher than a chest x‐ray.^11^ The risk of cancer from CT chest scans is 1 in 380 and 1 in 1020 in young females and males, respectively, with increased risk from recurrent scans due to cumulative radiation dose.^12^ Furthermore, the cost of a CT thorax is £147.34 according to the NHS reference cost 2021–2022.^13^ Avoiding unnecessary imaging reduces hospital expenses, particularly given the low incidence of pulmonary metastasis in this population.

Most abnormalities detected on the CT chests were indeterminate lung nodules, which required further surveillance or referral to the lung MDT. Given that the study period overlapped with the COVID‐19 pandemic, some of these incidental findings may have been related to imaging manifestations of COVID‐19.^14^ In this study, 14% of patients with cT1a renal masses had abnormalities detected on staging CT chest, with most findings being indeterminate pulmonary nodules. While COVID‐19 may have contributed to an increased detection of incidental nodules, there are no pre‐pandemic studies specifically demonstrating a lower incidence than in this patient population. Existing literature suggests that the detection rates of pulmonary nodules on CT chest vary widely depending on the studied population and imaging indication.^15^, ^16^, ^17^ Therefore, our 14% detection rate falls within the range reported in other clinical contexts, and we cannot definitively conclude that our findings were significantly influenced by the pandemic.

Moreover, three out of eight of the patients referred to the lung MDT required biopsies that confirmed benign nodules. These invasive investigations with their associated risks and complications could have been avoided since no metastasis secondary to renal cancer was identified. However, 1% of the cohort was found to have concurrent primary lung tumours. This is likely due to shared risk factors such as smoking history, age and family history.^18^

Targeted lung cancer screening with a single low‐dose CT in at‐risk populations has been shown to detect lung cancers in 4.7%,^19^ and a population‐based screening programme is currently being rolled out in the UK targeting smokers and ex‐smokers aged 55–74.^20^

There is currently no population‐based screening programme for kidney cancer. The Yorkshire Kidney Screening Trial assessed the feasibility of screening for kidney cancer in an at‐risk population of older smokers attending lung cancer screening by adding a non‐contrast upper abdominal CT. Only 0.25% were found to have histologically proven renal cancer,^21^ which is lower than the detection rate in our study.

Lastly, it is important to note that the recommendations on imaging for staging in T1a renal masses vary across guidelines. Whilst EAU guidelines recommend CT as the only modality for chest staging, American Urological Association (AUA) and American College of Radiology (ACR) recommends chest x‐ray in the first instance in a patient suspected with lower risk renal masses, with selective use of chest CT reserved for those with pulmonary symptoms or abnormal chest x‐ray or high risk renal cancer.^22^, ^23^ However, a study found that chest x‐rays are a low‐yield diagnostic tool for detecting lung metastasis in patients treated for T1a renal tumours, where only 1 of 3 pulmonary metastases were picked up with standard chest x‐ray surveillance.^24^

This study has several limitations. We did not systematically review follow‐up surveillance imaging for patients who did not receive a baseline CT chest. Given the retrospective nature of this study, it was difficult to discern the reasoning behind the decision for patients to have a CT chest at the first instance or as part of the first surveillance scan, as this is rarely documented on the MDT outcome forms available electronically. It could be that patients who have had a staging CT chest may have had different individual characteristics compared to those who did not have a CT chest at baseline; hence, the results obtained in this study are subject to selection bias. Lastly, our cohort in a large tertiary referral centre may not be representative of other centres and settings. To our knowledge, this is the first study to be conducted in this field in England. However, a retrospective cohort study in Scotland of 696 patients also concluded that patients with cT1a tumours could safely avoid CT chest, and a large cohort study in Korea restricted recommendations for CT Chest only in those with cT1b stage or above renal tumours.^25^, ^26^

In conclusion, the incidence of metastatic renal cell carcinoma in the thorax for newly diagnosed cT1a renal tumours is extremely low. In this study, baseline staging CT chest was inconsequential to the definitive management of 99% of patients diagnosed with a cT1a renal tumour, yet prompted further investigations in 14% of patients, the majority of which were ultimately benign. Non‐selective utilisation of CT chest frequently led to a diagnosis of indeterminate pulmonary nodules, with patients being over‐investigated with unnecessary further exposure to ionising radiation as a result of interval imaging. This approach is not only anxiety‐inducing for patients but leads to increased hospital visits, resource use and expenditure. Our results support the EAU recommendation that staging CT chest to assess for metastases could be omitted in patients with cT1a renal tumours. However, the detection of synchronous primary lung cancer in a subset of patients underscores the potential value of targeted lung cancer screening in high‐risk groups. Future research should focus on refining risk stratification to optimise imaging strategies, balancing early detection with the avoidance of unnecessary interventions.

## AUTHOR CONTRIBUTIONS

S. Ilangovan: *data collection*, *data analysis*, *manuscript writing/editing*. H. Warren: *project development*, *data analysis*, *manuscript writing/editing*. F. Sordelli: *manuscript writing/editing*. T. Paing: *data collection*. P. Phyo Tun: *data collection*. P. Patki: *project development*. F. Mumtaz: *project development*. R. Barod: *project development*. A. Bex: *project development*. MGB Tran: *project development*, *manuscript writing/editing*.

## CONFLICT OF INTEREST STATEMENT

The authors declare no conflict of interests that are relevant to this article's content.
